# A Long-Acting Combination Nerve Block in Rhinoplasty to Minimize Postoperative Opioid Use

**DOI:** 10.1093/asjof/ojag022

**Published:** 2026-02-24

**Authors:** Madison Mai-Lan Cheung, Anil R Shah

## Abstract

Minimizing the use of narcotics has been a primary concern in rhinoplasty in order to limit severe side effects and complications associated with opioid use, with addiction being of particular concern. The senior author previously described a bupivacaine nerve block technique in rhinoplasty that significantly reduced postoperative recovery times as well as narcotic, antiemetic, and benzodiazepine use. The aim of this study was to see whether a more potent nerve block composed of bupivacaine with the addition of dexmedetomidine and dexamethasone will result in the patient's avoidance of narcotics following rhinoplasty. A retrospective analysis was conducted on a total of 357 consecutive patients who underwent primary rhinoplasty with a single surgeon. Patients were administered an updated nerve block with a combination of bupivacaine, dexmedetomidine, and dexamethasone. The length of recovery time and use of postoperative medications were collected. In this cohort, 355 of 357 patients avoided postoperative opioids with a 99.4% success rate. No patients experienced side effects from the nerve block. The use of a combination nerve block with bupivacaine, dexmedetomidine, and dexamethasone can lead to successful reduction of postoperative opioid use. Future studies will compare the use of postoperative medications and duration of recovery time between patients who were administered this updated combination nerve block and those who did not receive the combination nerve block.

**Level of Evidence**: 4 (Therapeutic) 
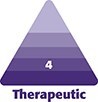

Minimizing the use of narcotics has been a primary concern in rhinoplasty in order to limit severe side effects and complications associated with opioid use, with addiction being of particular concern. Other complications include respiratory depression, dizziness, nausea, vomiting, constipation, and sedation.^1^ In rhinoplasty, an average of 28 opioid pills are prescribed to around 97.1% of patients.^2^ The average opioid consumption within 14 days following rhinoplasty is 6.15 pills.^3^

The senior author previously described a bupivacaine nerve block technique in rhinoplasty that significantly reduced postoperative narcotic, antiemetic, and benzodiazepine use.^4^ In the previous study, 42% of patients who were not given a bupivacaine nerve block required additional opioids to manage postoperative pain. Additionally, postoperative recovery times decreased by 74 min for patients who received a bupivacaine nerve block compared with those who did not receive a nerve block.

The incorporation of dexmedetomidine and dexamethasone can provide potential benefits to nerve blocks by lengthening the duration of the block. Dexamethasone has been shown to increase the duration of nerve block by 6.7 h.^5^ Another study has observed an extended duration of nerve block with the addition of dexmedetomidine to dexamethasone.^6^ In this case series, we attempt to establish whether a more potent nerve block composed of bupivacaine with the addition of dexmedetomidine and dexamethasone will result in the patient's avoidance of narcotics (Figure 1).

**Figure 1.**
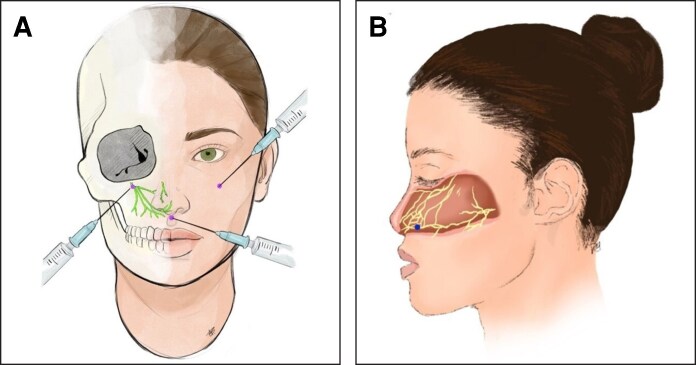
Locations of nerve block injections. (A) Bilateral infraorbital and subnasale locations. (B) Septum nerve block injection location.

## METHODS

A retrospective chart review of 357 consecutive patients who underwent primary rhinoplasty by the senior author at a single private institution between April 2024 and December 2024. In our study cohort, all patients underwent septoplasty and lateral osteotomies. Patients with a history of previous rhinoplasty, septoplasty, or taking additional pain medications were excluded. This study was approved by the Solutions Institutional Review Board (Shelton, CT; no. IORG0007116). Charts were reviewed for patient demographics (age, sex, and comorbidities) and opioid use following rhinoplasty. Postoperative opioid use was measured by the number of opioid prescriptions called in for the patient in the 1-week follow-up period. Descriptive statistics were conducted to analyze patient demographics and postoperative opioid use.

**Figure 2.**
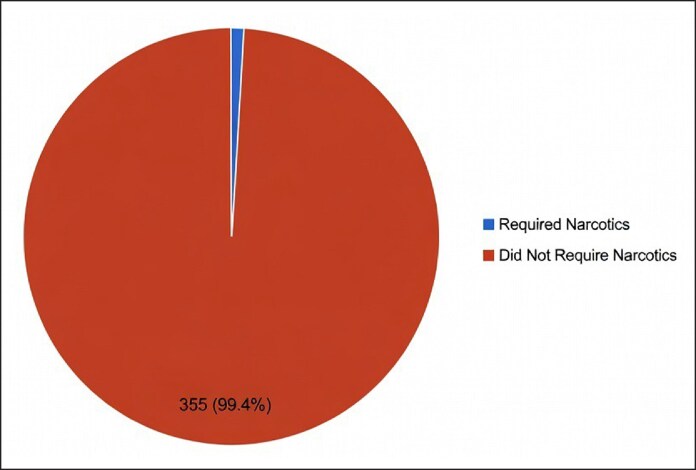
Postoperative narcotics requirement for patients given a combined nerve block.

Preoperative injections were performed using a combination injection of 1.0 cc of 0.25% bupivacaine, 0.5 cc of 100 μg/mL dexmedetomidine, and 0.5 cc of 4 mg/mL dexamethasone. The total solution of 2.0 cc was divided equally for injection in the bilateral infraorbital regions, along the inferior border of the septum, and along the columella in the subnasale region (Figure 2). These areas were chosen because they are consistently reported as primary sources of discomfort. These injections took place before the field block traditionally used in septorhinoplasty, which consists of 9 cc of local anesthesia of 1% lidocaine with 1:100,000 epinephrine injected throughout the soft tissues of the nose. The nerve blocks were used in combination with acetaminophen, gabapentin, and celecoxib, as has been previously established in ERAS protocols: 1000 mg oral acetaminophen, 200 mg of oral celecoxib, and 1200 mg oral gabapentin once preoperatively.^5^ An additional 1.5 cc total of the same nerve block was reapplied to the infraorbital nerves and subnasale region after the case was concluded. Patients' postoperative pain was managed with 1000 mg of acetaminophen every 4 to 6 h, 400 mg gabapentin twice daily, and 200 mg of celecoxib twice daily for 7 days. Prescriptions for opioids were also ordered to be filled as needed for pain that could not be adequately relieved. Assessment of pain requiring opioid use was observed by reviewing the patient's medical chart for whether opioid prescriptions were called in for the patient within the first week postoperatively.

## RESULTS

In this cohort, 357 patients were included. The mean age was 27.1 ± 8.3 years; the range was 18 to 71 years. The cohort was comprised of 89 (24.9%) male patients and 268 (75.1%) female patients. There were no patients with a history of diabetes, hypertension, tobacco use, depression, illicit drug use, or fibromyalgia. Approximately 15% of patients received internal nasal splints.

In total, 355 patients avoided postoperative opioids with a 99.4% success rate (Figure 2). Within the first week following their procedure, 2 patients requested the use of prescription opioids in the 72 h postoperative period. One patient had a history of narcotic use in the past. No cases of persistent analgesia, nerve pain, or systemic effects were reported.

## DISCUSSION

Opioids are commonly used to reduce pain after rhinoplasty. Most pain is found to be within the first 3 days after rhinoplasty, and analgesia is recommended for all patients during this time period.^7^ Patient satisfaction is better when pain is controlled, including better perception of functional improvement.^8^

However, opioids are often overprescribed, with patients using only 40% of prescribed pills for sufficient pain management.^9^ Access to overprescribed pills is known to lead to addiction and misuse, dispersion of opioids into the community, and the potential of further illicit drug or heroin use.^10^

In the current study, we found that 99.4% of patients avoided opioids following rhinoplasty when administered a combination nerve block consisting of bupivacaine, dexmedetomidine, and dexamethasone. The mechanism of action of nerve blocks includes preventing nerve impulses from propagating by closing sodium channels.^11^ The senior author previously observed bupivacaine nerve blocks decrease postoperative recovery times, additional opioid consumption, and antiemetic use.^4^ Furthermore, levobupivacaine nerve blocks to the infraorbital nerve, sphenopalatine ganglion, external nasal nerve, and central facial nerve are known to decrease postoperative pain and opioid requirements while increasing duration until first analgesia request.^12^

Dexamethasone helps to extend nerve block duration by 6.7 h and reduce postoperative pain when administered perineurally.^5^ Intravenous dexamethasone extends the duration of nerve block by 6 h when compared with placebo. However, there is no clinically significant difference in postoperative pain between intravenous and perineural dexamethasone. Dexmedetomidine has also been established as an effective adjuvant to peripheral nerve blocks in a variety of operations. In a meta-analysis, Abdallah and Brull reported dexmedetomidine extended the duration of sensory block by 150 min when administered intrathecally and 284 min when administered perineurally.^13^ Addition of dexmedetomidine is also associated with lower pain scores and decreased opioid use.^14^ There is evidence that a nerve block consisting of dexmedetomidine and dexamethasone combined improves duration when compared with placebo. Maagaard et al found that a dexmedetomidine–dexamethasone nerve block extended the duration of analgesia by 564 minutes with statistical significance.^6^ However, several studies have noted that there was no difference in the effects of the dexmedetomidine–dexamethasone combination compared with dexamethasone alone.^6^ The effectiveness of a combined dexmedetomidine–dexamethasone nerve block is still being studied.

Although the combination nerve block in this study was successful in minimizing postoperative opioid use, there is the potential risk of side effects. Side effects associated with local anesthetics are rare but include central nervous system toxicity and cardiovascular collapse.^15^ Nerve injury is another possible iatrogenic effect of nerve blocks and includes needle damage, rupture perineurium, reduced blood flow, and direct chemical injury to nerve.^16^ The rate of neuropathy after peripheral nerve block is <3:100, or 3%.^16^ In the current study, no patients experienced side effects related to nerve block administration.

The location of the nerve block sites was based on the experience of the senior author. Based on previous queries of patients in the postoperative recovery room about the location of pain, as well as where patients would react to pain under general anesthesia, the 3 locations (bilateral infraorbital regions, inferior border of the septum, and columella in the subnasale region) were chosen. The inferior border of the septum is highly sensitive and represents a confluence of nerves.^17^ The inferior aspect of the incision, as well as the infraorbital nerve, supplies sensation to 25.8 cm^2^ of the patient's face.^18^

There are several limitations to this study. The cohort consisted of patients at a single institution by a single surgeon. This can affect generalizability to a larger population, because there is variation in opioid prescribing protocols and surgeon techniques. There was no use of validated patient-reported outcome measures to assess patient pain. Additionally, as this was a case series, no control data was collected, and thus comparative statistics were not conducted and significance could not be concluded. The next phase of research will aim to comparatively analyze patients who were administered this updated combination nerve block and those who did not receive the combination nerve block to determine whether statistically and clinically significant differences exist between pain scores at various time intervals, medication use, and recovery time.

## CONCLUSIONS

Longer-lasting injections can be helpful in reducing opioid consumption. Three hundred and fifty-five of 357 patients avoided opioids with a 99.4% success rate and no reported side effects. The combination nerve block of bupivacaine, dexmedetomidine, and dexamethasone can lead to reduced opioid use after septorhinoplasty.
